# Retention Challenges in Opioid Use Disorder Treatment: The Role of Comorbid Psychological Conditions

**DOI:** 10.5811/westjem.52941

**Published:** 2026-01-03

**Authors:** David C. Seaberg

**Affiliations:** US Acute Care Solutions, Canton, Ohio

Dear Editor:

We would like to thank Dr. Shu Yuan and colleagues for their insightful letter. The treatment of patients with opioid use disorder (OUD) presents a myriad of complicating factors, making both successful treatment and retention particularly challenging. They note that patients may be more likely to remain in treatment if they enter a program de novo, rather than following relapse or overdose. While our study did not specifically evaluate this factor, it is a reasonable assumption that warrants further exploration.

Over the five-year period of our investigation, we observed a decline in enrollment for medication for opioid use disorder (MOUD) treatment.^1^ This trend may reflect a growing “distrust” in the treatment system among patients or, as we hypothesize, may indicate that MOUD alone is not universally effective for all individuals. Importantly, our study highlighted the role of psychosocial factors in risk stratification of MOUD patients, suggesting that these factors may be critical for predicting treatment adherence and outcomes.

We hope that future research will focus on integrating psychosocial determinants into OUD care strategies, thereby improving retention and treatment success for this complex and vulnerable patient population.
